# Limitation of the Benefit of Amphotericin-Flucytosine Combination Therapy in Patients With Lower Conscious Level: An Ecological Fallacy?

**DOI:** 10.1093/ofid/ofv069

**Published:** 2015-05-14

**Authors:** Marcel Wolbers, Jeremy N. Day

**Affiliations:** 1 Oxford University Clinical Research Unit, Hospital for Tropical Diseases, Ho Chi Minh City, Vietnam; 2 Centre for Tropical Medicine, Nuffield Department of Clinical Medicine, University of Oxford, United Kingdom

To the editor—We were interested to read Campbell and colleagues' timely network meta-analysis of antifungal therapies for HIV-associated cryptoccocal meningitis,recently published in the journal [1]. One of the authors' main conclusions is that the benefit of combination therapy with amphotericin B and flucytosine seems to be limited to individuals with altered consciousness at treatment initiation, and the discussion refers to our recent trial [2] to support this claim. However, it is important to note that the meta-analysis was based on study-level data only, and the covariate that was actually investigated was not whether an individual patient had altered consciousness, but whether the study included subjects with altered consciousness. Interpretation of population characteristics on the patient level is prone to the ecological fallacy, a well known risk of meta-regression analyses [3]. For example, our trial both included a relatively high proportion of subjects with altered consciousness and reported a significant mortality benefit of amphotericin B plus flucytosine compared with amphotericin B monotherapy [2]. However, it would be wrong to conclude that this indicates that the mortality benefit mainly applies to subjects with altered consciousness. Indeed, a subgroup analysis of our trial indicates that there is no evidence that the treatment effect depends on the Glasgow coma score (GCS), and, if anything, the subgroup effect in subjects with normal consciousness (GCS = 15) is even more convincing than in subjects with a GCS < 15 (Table 1).

**Table 1.** Mortality at Day 14, Week 10 (Coprimary Endpoints) and Month 6 Overall and by GCS Score at Enrollment in the Vietnam Trial [2]

	Amphotericin B (N = 99) : Deaths/n (Mortality*)	Amphotericin B and Flucytosine (N = 100): Deaths/n (Mortality*)	Comparison: HR (95% CI), *P* Value	Test for Heterogeneity: *P* Value
Death by day 14				
All patients	25/99 (25%)	15/100 (15%)	0.57 (.30–1.08), *P* = .08	
GCS = 15	14/66 (21%)	5/67 (7%)	0.31 (.11–.87), *P* = .03	
GCS < 15	11/31 (35%)	10/32 (31%)	0.90 (.38–2.12), *P* = .81	.12
Death by week 10				
All patients	44/99 (44%)	30/100 (31%)	0.61 (.38–.97), *P* = .04	
GCS = 15	24/66 (36%)	15/67 (23%)	0.53 (.28–1.00), *P* = .05	
GCS < 15	20/31 (65%)	15/32 (48%)	0.72 (.37–1.40), *P* = .33	.57
Death by month 6				
All patients	53/99 (54%)	34/100 (35%)	0.56 (.36–.86), *P* = .01	
GCS = 15	32/66 (48%)	18/67 (27%)	0.46 (.26–.83), *P* = .01	
GCS < 15	21/31 (68%)	16/32 (51%)	0.71 (.37–1.35), *P* = .30	.42

Abbreviations: CI, confidence interval; GCS, Glasgow coma score; HR, hazard ratio (based on Cox regression).

*GCS at baseline was missing for 3 subjects. Mortality was estimated with the Kaplan-Meier estimator. Heterogeneity was tested with an interaction test between the subgrouping variable (GCS = 15 vs <15) and the treatment allocation. The amphotericin B plus fluconazole arm is not reported here.

More generally, we believe that our trial supports the use of amphotericin B plus flucytosine in populations with severe disease, as frequently seen in resource-limited settings, and we were pleased to read that the authors also endorse advocacy efforts to increase the availability of flucytosine in such settings. Although the presented network meta-analysis provides valuable aggregate evidence, it is our opinion that the evidence is not sufficiently strong to override the conclusions from our large and well designed trial, given the limitations cited by the authors regarding substantial heterogeneity between the included study populations, small sample sizes of many of the included studies, the inclusion of nonrandomized studies, and well recognized issues related to indirect comparisons [4]. However, we agree with the authors that the value of additional flucytosine in patients with less advanced disease than seen in our trial has not been established. Finally, we strongly agree with the author's conclusion that further well designed and adequately powered studies using clinical endpoints are required to improve the evidence base for determining the optimal treatment of patients with HIV-associated cryptococcal meningitis. It is important that these studies do not arbitrarily exclude “difficult to study” patients, such as those with severe disease, to ensure that study populations are representative of the general patient population and study findings are generalizable.

## References

[1] Campbell JI Kanters S Bennett JE Comparative effectiveness of induction therapy for human immunodeficiency virus-associated cryptococcal meningitis: a network meta-analysis. Open Forum Infect Dis 2015; 2 10.1093/ofid/ofv010 10.1093/ofid/ofv010 PMC4438891 26034761

[2] Day JN Chau TT Wolbers M Combination antifungal therapy for cryptococcal meningitis. N Engl J Med 2013; 368:1291–302 23550668 10.1056/NEJMoa1110404 PMC3978204

[3] Thompson SG Higgins JP How should meta-regression analyses be undertaken and interpreted? Stat Med 2002; 21:1559–73 12111920 10.1002/sim.1187

[4] Salanti G Indirect and mixed-treatment comparison, network, or multiple-treatments meta-analysis: many names, many benefits, many concerns for the next generation evidence synthesis tool. Res Synth Methods 2012; 3:80–97 26062083 10.1002/jrsm.1037

